# Natural Selection of Human Embryos: Decidualizing Endometrial Stromal Cells Serve as Sensors of Embryo Quality upon Implantation

**DOI:** 10.1371/journal.pone.0010258

**Published:** 2010-04-21

**Authors:** Gijs Teklenburg, Madhuri Salker, Mariam Molokhia, Stuart Lavery, Geoffrey Trew, Tepchongchit Aojanepong, Helen J. Mardon, Amali U. Lokugamage, Raj Rai, Christian Landles, Bernard A. J. Roelen, Siobhan Quenby, Ewart W. Kuijk, Annemieke Kavelaars, Cobi J. Heijnen, Lesley Regan, Jan J. Brosens, Nick S. Macklon

**Affiliations:** 1 Department of Reproductive Medicine and Gynecology, University Medical Center Utrecht, Utrecht, The Netherlands; 2 Institute of Reproductive and Developmental Biology, Imperial College London, Hammersmith Hospital, London, United Kingdom; 3 Department of Epidemiology and Population Health, London School of Hygiene & Tropical Medicine, London, United Kingdom; 4 Nuffield Department of Obstetrics and Gynecology, University of Oxford, Women's Centre, John Radcliffe Hospital, Oxford, United Kingdom; 5 Department of Obstetrics and Gynecology, the Whittington Hospital NHS Trust, London, United Kingdom; 6 Faculty of Veterinary Medicine, Utrecht University, Utrecht, The Netherlands; 7 Department of Reproductive and Developmental Health, Liverpool Women's Hospital, University of Liverpool, Liverpool, United Kingdom; 8 Laboratory of Psychoneuroimmunology, University Medical Center Utrecht, Utrecht, The Netherlands; 9 Division of Developmental Origins of Health and Disease, Princess Anne Hospital, University of Southampton, Southampton, United Kingdom; Indiana University, United States of America

## Abstract

**Background:**

Pregnancy is widely viewed as dependent upon an intimate dialogue, mediated by locally secreted factors between a developmentally competent embryo and a receptive endometrium. Reproductive success in humans is however limited, largely because of the high prevalence of chromosomally abnormal preimplantation embryos. Moreover, the transient period of endometrial receptivity in humans uniquely coincides with differentiation of endometrial stromal cells (ESCs) into highly specialized decidual cells, which in the absence of pregnancy invariably triggers menstruation. The role of cyclic decidualization of the endometrium in the implantation process and the nature of the decidual cytokines and growth factors that mediate the crosstalk with the embryo are unknown.

**Methodology/Principal Findings:**

We employed a human co-culture model, consisting of decidualizing ESCs and single hatched blastocysts, to identify the soluble factors involved in implantation. Over the 3-day co-culture period, approximately 75% of embryos arrested whereas the remainder showed normal development. The levels of 14 implantation factors secreted by the stromal cells were determined by multiplex immunoassay. Surprisingly, the presence of a developing embryo had no significant effect on decidual secretions, apart from a modest reduction in IL-5 levels. In contrast, arresting embryos triggered a strong response, characterized by selective inhibition of IL-1β, -6, -10, -17, -18, eotaxin, and HB-EGF secretion. Co-cultures were repeated with undifferentiated ESCs but none of the secreted cytokines were affected by the presence of a developing or arresting embryo.

**Conclusions:**

Human ESCs become biosensors of embryo quality upon differentiation into decidual cells. In view of the high incidence of gross chromosomal errors in human preimplantation embryos, cyclic decidualization followed by menstrual shedding may represent a mechanism of natural embryo selection that limits maternal investment in developmentally impaired pregnancies.

## Introduction

Pregnancy depends on intimate interactions between a developmentally competent embryo and a receptive endometrium. The endometrial luminal epithelium, the primary barrier for implantation, is not normally receptive and must transiently acquire this phenotype to allow first apposition and then attachment of the blastocyst [1]. In humans, the putative ‘implantation window' opens 6 days after the postovulatory progesterone surge and is thought to last no longer than 2–4 days [2]. Once the luminal epithelium is breached, most invading mammalian embryos elicit a decidual response, characterised by transformation of stromal fibroblasts into secretory, epitheloid-like decidual cells, influx of specialized uterine immune cells and vascular remodelling [3], [4]. In the human situation, however, this decidual response is primarily under maternal control and initiated in the mid-secretory phase of each cycle, irrespective of whether pregnancy has occurred or not.

Spontaneous decidualization of the stromal compartment in the absence of pregnancy invariably causes menstruation, a rare biological phenomenon confined to a handful of species [5]. The reason is that once the endometrium undergoes a decidual response, the integrity of the tissue becomes inextricably dependent upon continuous progesterone signalling. In the absence of pregnancy, declining progesterone levels triggers a switch in the secretory repertoire of decidual stromal cells, now characterized by expression of pro-inflammatory cytokines, chemokines and matrix metalloproteinases, which activates a sequence of events leading to tissue breakdown and menstrual shedding [6], [7].

Another feature of human reproduction is that it is remarkable inefficient [8]. Monthly fecundity rates in fertile couples average around 20%, which is disappointingly low compared to many species [5]. While this lack of intrinsic reproductive efficacy may reflect a multitude of complex social and biological factors, for example the loss of estrous behaviour and concealed ovulation, it is foremost attributable to the high prevalence of chromosomal abnormalities in human embryos, which limits their developmental potential and accounts for the age-dependent decline in fertility [9], [10]. The emergence of array-based technologies that allow genome-wide screening of individual cells has revealed that the frequency and complexity of chromosomal aberrations are much higher in early human embryos than hitherto appreciated [11]. Using this approach, a recent study demonstrated that less than 10% of cleavage-stage IVF embryos, obtained from fertile women under the age of 35, have a normal karyotype in all blastomeres, approximately half have no normal cells at all, and the remainder are mosaic for large-scale structural chromosomal imbalances, caused predominantly by mitotic non-disjunction [12]. While these observations suggest that not all chromosomal rearrangements in human preimplantation embryos compromise subsequent viability, they also imply that selection mechanisms must exist that limit maternal investment in a defective conceptus.

Human implantation sites are for ethical reasons inaccessible *in vivo*. Our understanding of early pregnancy events is therefore largely based on animal experiments, especially knockout studies in mice, and on gene expression studies in the human endometrium, aimed at identifying those factors that underpin the transient receptive phenotype [1], [13]. These studies indicate that implantation requires activation of evolutionarily conserved endometrial transcription factors (e.g. HOXA10, STAT3, p53) [14]–[17], expression of key cell adhesion molecules and their ligands (e.g. αvβ3 integrin, trophinin, L-selectin ligand) [18]–[20], and several growth factors and cytokines [1], all of which are essential for coordinated cross-talk with the implanting embryo, at least in mice.

In this study, we set out to characterize key soluble factors involved in crosstalk between endometrial stromal cells (ESCs) and an interacting embryo, using a validated human co-culture model [21]. Based on the current implantation paradigm, we anticipated that only developing embryos would express the appropriate molecular repertoire to engage with endometrial cells. Instead, we found that ESCs selectively recognize and respond to the presence of a developmentally impaired embryo but only upon differentiation into decidual cells.

## Results

**Figure 1:**
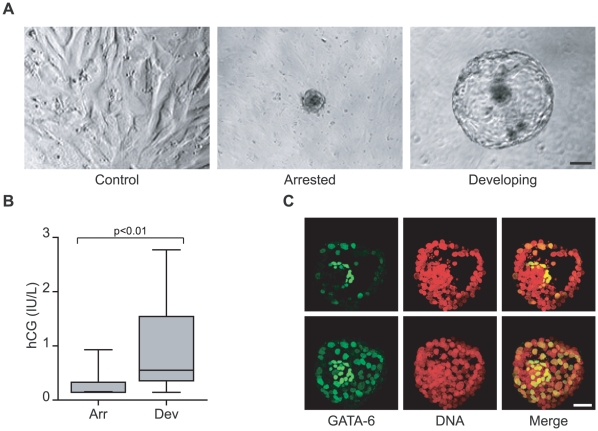
Human embryo development in co-culture. (*A*) Phase contrast images of decidualizing ESCs alone (control; left) or in the presence of an arrested (middle) or developing human embryo (right) (scale bar  = 100 µm). (*B*) Arrested embryos (Arr) express significantly less hCG than developing embryos (Dev). Secreted hCG levels were assayed after 72 hours of co-culturing human embryos and decidualizing ESCs (*P*<0.01). (*C*) Formation of primitive endoderm in developing human embryos co-cultured with decidual ESCs. Optical cross-sections through a day 8 embryo, cultured first on a decidualizing ESC monolayer for 72 hours, demonstrates the presence of GATA-6 positive cells aligning predominantly to the blastocoelic surface of the inner cell mass, which corresponds to the location of the primitive endoderm (top panel). The lower panel represents z-projection of all the images in the stack from a top to bottom scan through the embryo (scale bar  = 50 µm).

Implantation is thought to be dependent on an intimate dialogue between the blastocyst and uterine tissues, mediated by locally secreted factors [1], yet the nature of this crosstalk in humans is unknown. To examine embryo-endometrial interactions, we first established 41 co-cultures in 16 mm wells, each with a single hatched blastocyst seeded onto a confluent monolayer of ESCs decidualized for 5 days with 8-Br-cAMP and the progestin medroxyprogesterone acetate (MPA), a widely used protocol that elicits a differentiation response similar to that observed *in vivo* [22], [23]. Control cultures received no blastocysts. Based on morphological criteria, 11 embryos developed normally beyond the expanding blastocyst stage over the 3-day co-culture period while 30 embryos arrested (Fig. 1A). Analysis of the culture supernatants further showed that developing embryos produced significantly higher human chorionic gonadotropin (hCG) levels (Fig. 1B). Moreover, confocal microscopy demonstrated that GATA-6, a transcription factor that marks the formation of the primitive endoderm [24], was selectively expressed at the blastocoelic side of the inner cell mass of co-cultured competent embryos, indicating that the *in vitro* conditions did not interfere with the normal embryonic developmental trajectory (Fig. 1C).

**Figure 2:**
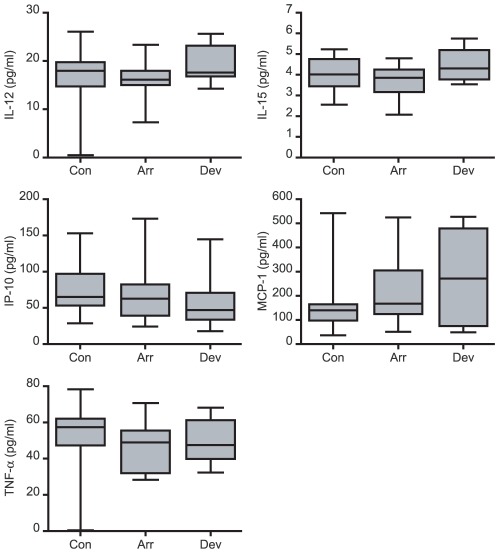
Secreted decidual cytokines not regulated upon embryo co-culture. Primary ESCs were first decidualized for 5 days and then co-cultured with human embryos or not (control cultures, Con). Over the 72-hour co-culture period, 30 embryos arrested (Arr) whereas 11 continued to develop normally (Dev). Analysis of the culture supernatants revealed that the presence of an arresting or developing human embryo had no significant impact on the secretion of the indicated factors (*P*>0.05).

**Figure 3:**
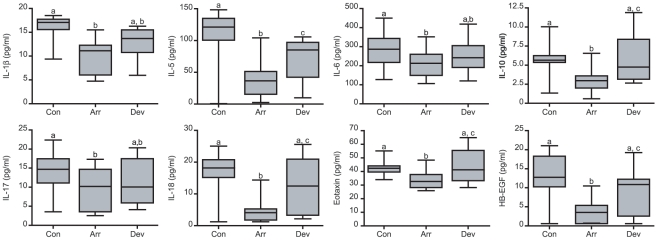
Developmentally impaired human embryos inhibit the secretion of selective implantation modulators by decidualizing ESCs. Primary ESCs were first decidualized for 5 days and then co-cultured with human embryos or not (control cultures, Con). Over the 72-hour co-culture period, 30 embryos arrested (Arr) whereas 11 continued to develop normally (Dev). Analysis of the culture supernatants revealed that the presence of an arresting embryo inhibited the secretion of the indicated factors. The letters above the box plots indicate significant differences between groups. *P*<0.01 for all comparisons except for IL-6 and IL-17 (*P*<0.05).

**Figure 4:**
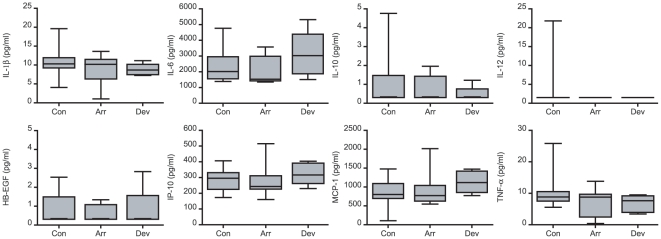
The human embryo does not elicit a secretory response in undifferentiated endometrium. Undifferentiated primary ESCs were co-cultured with embryos or not (control cultures, Con). Over the 72-hour co-culture period, 15 embryos arrested (Arr) whereas 6 continued to develop normally (Dev). Co-culture with either an arrested or developing embryo had no impact on the secreted levels of the indicated factors (*P*>0.05). The concentrations of IL-5, -12, -15, -17, -18, and eotaxin in culture supernatants of undifferentiated ESCs were below the level of detection.

Next, we used a multiplex immunoassay to determine the levels of 14 secreted cytokines, chemokines and growth factors, all of which have putatively been implicated in the process of implantation [25]. Decidualizing ESCs produced all mediators with the exception of IFN-γ. The presence of an embryo had no discernable effect on IL-12, -15, TNF-α, MCP-1 or IP-10 production (Fig. 2). In contrast, decidual cells in co-culture secreted significantly lower levels of IL-1β, -6, -10, -17, -18, eotaxin, and HB-EGF but only in the presence of an arresting embryo (Fig. 3). IL-5 secretion, however, was inhibited irrespective of embryo quality, although more so in response to a developmentally impaired embryo. The co-cultures were repeated with undifferentiated ESCs, which lack the pronounced secretory phenotype of decidual cells. Nevertheless, 8 cytokines, including IL-1β, -10, and HB-EGF, accumulated in the culture supernatants over 3 days but none were affected by the presence of a developing or arresting embryo (Fig. 4). These data suggest that abnormal human embryos engage in intense signalling upon implantation, which in turn triggers a profound regional endometrial response but only following adequate decidualization of the stromal compartment.

## Discussion

Unravelling the mechanisms that control implantation is paramount to improving reproductive outcome, especially in couples suffering from infertility or recurrent pregnancy loss. Gene deletion studies leading to reproductive failure in mice have been hugely important in defining factors essential for embryo-endometrial interactions [26]–[28]. While it seems reasonable to assume that many of these implantation factors will be evolutionary conserved, especially in mammals where pregnancy depends on invasion of maternal tissues, it should also be acknowledged that there are important interspecies differences. For example, a large number of mammals, including mice, are capable of delaying implantation by temporarily suspending embryo development [29]. This process, termed ‘diapause’, is reversed when the endometrium signals an optimal metabolic and hormonal intrauterine milieu. There is, however, no evidence that human embryos are capable of delaying implantation while awaiting the right maternal signals. Conversely, and as mentioned before, it is the embryo that triggers a decidual response in mice and most other species, yet initiation of this process is uncoupled from embryonic signals in humans [3]. Finally, gross embryonic chromosomal abnormalities are rare in most mice strains, which means that little if anything is known about the fate of developmentally impaired embryos.

Our co-culture experiments suggest that spontaneous decidualization of human endometrium, which in the absence of a viable pregnancy inevitably leads to menstruation, serves as a mechanism for embryo quality control. This supposition is based on the observation that decidualized but not undifferentiated ESCs selectively recognize the presence of a developmentally impaired embryo and respond by inhibiting the secretion of key implantation mediators (e.g. IL-1β and HB-EGF) and immunomodulators (e.g. IL-5, -6, -10, -11, -17, and eotaxin). Considering that single human embryos were co-cultured with at least 50,000 ESCs, the magnitude of the maternal response is formidable. Apart from a modest reduction in IL-5 secretion, ESCs, decidualized or not, appear largely indifferent to the presence of a developing embryo. It is possible of course that the molecular dialogue with a competent conceptus is either more selective or confined to decidual cells in the immediate vicinity and, hence, insufficient to significantly impact on the total secreted levels of these factors.

The importance of the endometrial stromal compartment in regulating early human implantation events is further illustrated by recent studies, demonstrating that differentiating ESCs acquire a motile and invasive phenotype in response to embryonic trophoblast signals [21], [30]. In other words, rather than being passively invaded, these observations suggest that decidualizing ESCs actively encapsulate the early human conceptus. If so, the phenomenal response of decidual cells to a developmentally impaired embryo could represent a mechanism for controlled embryo disposal, mediated by induction of menstruation-like tissue breakdown and shedding. Intriguingly, women with early pregnancy losses bled less than their typical menses following pregnancies of very short duration and more than usual for the pregnancies lasting approximately six weeks. This indicates that menses might reflect endometrial factors associated with early pregnancy loss [31], [32].

Our observations raise a number of important but as yet unanswered questions. First, the nature of the embryonic signals capable of modulating the decidual secretome remains elusive, although it is tempting to speculate that they are generated in response to the much higher metabolite or nutrient turnover rates that characterize arresting embryos [33]. Secondly, while we show that ESCs become biological sensors of embryo quality upon decidualization, the underpinning mechanism and that of the subsequent secretory response are unclear. Decidualized but not undifferentiated ESCs form dense intercellular connections, such as adherens and tight junctions [34], which may be essential to propagate embryo-derived factors, such as metabolites, throughout the culture in a receptor-independent manner. Thirdly, whether or not the developmentally impaired embryos in our co-culture system retain an invasive phenotype remains to be determined. Conversely, it is also important to ascertain if the nature and magnitude of the decidual response correlates somehow with the frequency and complexity of gross chromosomal errors in the co-cultured blastocyst. A finely tuned, tailored decidual response is not beyond the realm of possibilities as a recent study in the bovine demonstrated that endometrial gene expression varies dramatically in response to implantation of an embryo conceived *in vivo*, after IVF or following somatic cell nuclear transfer [35]. Finally, and most importantly, clinical studies are required to determine the extent to which impaired decidualization and embryo recognition underpins miscarriage and other pregnancy complications. The accompanying paper by Salker *et al*. in this journal describes our attempt at addressing this important question.

In summary, rather than being biologically silent or inert, our data suggest that developmentally impaired human embryos can trigger a formidable maternal response, which requires decidual transformation of the endometrium. From an evolutionary perspective, our findings are in agreement with the genetic conflict theory of pregnancy [36], [37], which predicts that changes in the embryonic genome are opposed by maternal countermeasures. Within this context, we propose that the emergence of cyclic decidualization of the human endometrium, which is inextricably linked to menstruation, is an adaptive response to the high incidence of chromosomal instability in human embryos. If correct, our findings predict that failure of the endometrium to express an adequate decidual phenotype disables natural embryo selection upon implantation and causes subsequent pregnancy failure.

## Materials and Methods

### Ethics statement and patient selection

This study was approved by the Medical Review Ethics Committee University Medical Center Utrecht and the Central Committee on Research inv. Human Subjects in The Netherlands (NL 12481.000.06). Written informed consent was obtained from all participating subjects, either for the use of supernumerary cryopreserved embryos or endometrial samples. All patients were investigated according to the standard clinic protocols, but the outcome of these routine investigations was not taken in account in either the recruitment into this study or in the analysis of the data.

### Embryo collection and co-culture

Ovarian stimulation, ovulation induction, oocyte retrieval and IVF/ICSI were performed according to standard clinical protocols. Supernumerary, good quality embryos were subsequently cryopreserved as previously described [38]. For this study, 140 day 4 embryos from 39 patients were thawed, taken through consecutive washes of 1.25, 1.00, 0.75 and 0.375 mol/l DMSO for 5 minutes each, then transferred to Human Tubal Fluid (HTF) culture medium supplemented with 10% GPO (human plasma solution; CLB, The Netherlands), overlaid with 1 ml of light paraffin oil (Irvine Scientific, Santa Ana, USA), and cultured until day 5. Sixty-two embryos from 27 patients survived the thawing procedure and extended culture period. These embryos were subjected to 0.1% Pronase/10% GPO treatment to remove the zona pellucida.

For the co-culture experiments, primary ESCs were purified, as previously described [22], from a single proliferative phase biopsy sample obtained from a patient with no uterine pathology or a history of RPL. The culture was expanded and the cells frozen at −80°C in aliquots, which were subsequently thawed consecutively to ensure identical conditions in all co-culture experiments. Thawed ESCs were seeded into 16 mm wells (0.5×10^5^ cells per well) in DMEM/F12 complete medium and grown until confluence. Decidualization was induced by the addition of 0.5 mM of 8-bromoadenosine 3′,5′-cyclic monophosphate (8-Br-cAMP; Sigma, UK) and 1 µM medroxyprogesterone acetate (MPA; Sigma, UK). This medium was changed every 48 hours. Individual blastocysts were then seeded onto a confluent layer of either undifferentiated ESCs or cells first decidualized for 5 days. Co-cultures were maintained in 250 µl of DMEM/F-12 complete medium for 72 hours. At the end of the co-culture period, the embryos were assessed for the quality of development. Culture medium was collected and stored at −80°C. ESC viability, assessed by trypan blue exclusion, was >90% for all cultures.

### Multiplex immunoassay and hCG measurements

The supernatant samples were analysed using a multiplex immunoassay capable of detecting interleukin (IL)-1β, IL-5, IL-6, IL-10, IL-12, IL-15, IL-17, IL-18, tumour necrosis factor α (TNF-α), interferon γ (IFN-γ), monocyte chemotactic protein 1 (MCP-1), chemokine (C-X-C motif) ligand 10 (IP-10), eotaxin [also known as chemokine (C-C motif) ligand 11 or CCL11] and heparin-binding EGF-like growth factor (HB-EGF) [25]. Antibodies were covalently coupled to the microspheres and the assay was carried out as described previously [25], [39]. Co-culture supernatants were analysed for hCG (human chorionic gonadotropin) production by the embryo using the Immulite 1000® immunoanalyser (Siemens). The functional sensitivity of the assay was 0.06 mU/mL with 20% coefficient of variation [25].

### GATA6 immunostaining and confocal imaging

Co-cultured day 8 human blastocysts were washed in PBS and fixed for 10 minutes at room temperature in 4% formaldehyde. After fixation, embryos were washed in 0.1% triton x-100 in PBS (PBST) and subsequently permeabilized in 0.5% triton x-100 in PBS for 15–30 minutes at room temperature (RT). Blocking was performed by incubating the embryos in 10% fetal calf serum in PBST (blocking solution) for 1 hour at RT followed by overnight incubation at 4°C in primary antibody (rabbit anti-GATA6; SC9055, Santa Cruz Biotechnology, Santa Cruz, US), diluted in blocking solution. The next day, the embryos were washed in PBST and transferred to blocking solution containing Alexa fluor conjugated secondary antibodies (Molecular probes, Invitrogen, Venlo, the Netherlands). After 1 hour embryos were washed, counterstained with TOPRO-3 (Molecular Probes, Invitrogen) and mounted in Vectashield mounting medium (Brunschwig Chemie, Amsterdam, the Netherlands). Fluorescent signals were visualized using a confocal laser scanning microscope.

### Statistical analyses

For multiple comparisons, ANOVA test with Bonferroni correction was used. *P*<0.05 was considered significant.
